# Moving beyond Karnofsky and ECOG Performance Status Assessments with New Technologies

**DOI:** 10.1155/2016/6186543

**Published:** 2016-03-15

**Authors:** Ciara M. Kelly, Armin Shahrokni

**Affiliations:** ^1^Gastrointestinal Oncology Service, Department of Medicine, Memorial Sloan Kettering Cancer Center, New York, NY 10065, USA; ^2^Geriatrics Service, Department of Medicine, Memorial Sloan Kettering Cancer Center, New York, NY 10065, USA

## Abstract

Progress in cancer research is coupled with increased treatment complexity reliant upon accurate patient selection. Oncologists rely upon measurement instruments of functional performance such as the Karnofsky or Eastern Cooperative Oncology Group Performance Status scales that were developed over fifty years ago to determine a patient's suitability for systemic treatment. These standard assessment tools have been shown to correlate with response to chemotherapy, chemotherapy tolerability, survival, and quality of life of cancer patients. However, these scales are subjective, subject to bias and high interobserver variability. Despite these limitations important clinical decisions are based on PS including eligibility for clinical trials, the “optimal” therapeutic approach in routine practice, and the allocation of healthcare resources. This paper reviews the past, present, and potential future of functional performance status assessment in an oncology setting. The potential ability of electronic activity monitoring systems to provide an objective, accurate measurement of patient functional performance is explored. Electronic activity monitoring devices have the potential to offer positive health-related opportunities to patients; however their introduction to the healthcare setting is not without difficulty. The potential role of this technology in healthcare and the challenges that these new innovations pose to the healthcare industry are also examined.

## 1. Introduction

We have witnessed significant advances in the fields of molecular oncology, cancer genomics, chemotherapy, and immunotherapy. The armamentarium of systemic anticancer therapies is expanding. This success is often coupled with increased treatment complexity reliant upon appropriate patient selection. For example, triplet chemotherapy is now a standard first-line option for advanced pancreas cancer reserved only for the “fit” individual [1].

“Personalized cancer care” is the phrase commonly used to describe the modern day treatment approach towards cancer. This model of care focuses primarily on identifying the molecular profile of the tumor and using this information to guide therapeutic decisions. However, despite the suggestion in its name, this model too often takes the individual out of the equation. Optimal “personalized cancer care” encompasses two key elements: (i) the molecular and genetic composition of the tumor and/or the individual and (ii) the patient's general biopsychosocial make-up, inclusive of his or her overall physical and emotional fitness and frailty. However, research and development in these two areas have been unequal. Substantial progress has been made from a molecular oncology and translational research perspective. Research focusing on patient-related, biopsychosocial factors and their influence on cancer outcomes has been limited, however. For example, oncologists still rely upon measurement tools of functional performance such as the Karnofsky or Eastern Cooperative Oncology Group Performance Status scales that were developed over fifty years ago to determine a patient's suitability for systemic treatment.

Patients with a poor performance status (PS) are associated with increased risk for chemotherapy toxicity and poor outcomes compared to patients with better performance status [2]. Accurate scoring of performance status is of critical importance because many clinical decisions are based on PS including the planning, randomization, eligibility for and evaluation of clinical trials, the “optimal” therapeutic approach in routine clinical practice, and the allocation of healthcare resources such as those of palliative home care agencies. Improving our ability to objectively and accurately assess patient fitness for cancer treatments may lead to better selection of therapeutic approaches for patients, reduce toxicity, and improve quality of life during cancer treatment. Optimal selection of therapeutic strategies for patients should help reduce the burden on healthcare resources with a resultant positive impact on the cancer care budget. In this paper we will review the past, present, and potential future of functional performance status assessment in an oncology setting.

## 2. Standard Measurement Instruments of Performance Status Used in Oncology

In general, physicians incorporate the findings of the patient history and physical examination to create a picture of a patient's health and functional status. The oncology community also uses additional tools to assess performance status or fitness for cancer treatments. The two most commonly used measurement instruments include (i) the Eastern Cooperative Oncology Group Performance Status (ECOG PS) and (ii) Karnofsky Performance Status (KPS) scales.

KPS was the first performance status scale described in 1949 [3]. Using this scale each patient is allocated a score on a linear scale between 0 (dead) and 100 (normally active, without evidence of disease), summarizing their ability to perform daily activities and the level of assistance they require in order to do so [4]. In 1960, ECOG introduced the ECOG PS scale with only six points, ranging from 0 (fully active) to 5 (dead) [5]. The two scales have been shown to be interchangeable, although the ECOG scale is often preferred for its simplicity and intraobserver reproducibility [6].

## 3. Correlation of Performance Status (PS) with Outcome Measures in Cancer

PS assessment has been shown to correlate with survival in many cancer forms [7, 8]. For example, in a study of 3825 patients with metastatic colorectal cancer receiving 5-fluorouracil, ECOG PS of 0 and 1 was associated with a longer duration of survival compared to ECOG PS >1 [9]. In the setting of non-small-cell lung cancer patients receiving first-line, doublet, platinum-based chemotherapy regimens patients with an ECOG PS of 2 had a significantly worse survival outcome than patients with an ECOG PS of 0 or 1. In fact the survival rate at one year for a patient with an ECOG PS of 0 was twice that of an individual with an ECOG PS of 2 (40 versus 19%, *p* < 0.001) [10].

PS was also shown to be predictive of both overall and complete response to cisplatin-based chemotherapy in the setting of metastatic urothelial cancer [11]. Clinical trials typically enroll less than 10% of patients with PS of 2. Pooled efficacy and safety data from nine clinical trials of patients with metastatic colorectal cancer examined the effect of PS on outcome measures including chemotherapy toxicity and 60-day all-cause mortality. Patients with PS 2 experienced significantly higher rates of grade ≥3 nausea and vomiting and 60-day all-cause mortality compared to patients with PS 0 or 1 [2]. An association has also been found between performance status and quality of life. Decreases in quality of life among stage IV lung cancer patients as assessed by the functional living index-cancer scale correlated with both a decline in PS and poor PS [12].

## 4. Limitations of the Standard Measurement Instruments of Performance Status Routinely Used by Oncologists

Although standard measurement instruments of PS are simple and useful, they are subject to bias and limitations. Reliability relates to the degree of confidence that we have in the individual measurements and the concordance between observers and is often described as intra- or interobserver variability. The literature reports conflicting data with respect to the reliability of PS measurements with variable levels of interobserver agreement [13, 14]. One study examined interobserver agreement when patients' PS was rated, using both KPS and ECOG PS scales, independently by a clinical oncologist, a ward resident medical officer, a principal training nurse, and the patient themselves [6]. The level of agreement was higher for the ECOG PS scale than the KPS scale. This result can be simply explained by the fact that the ECOG PS scale has a smaller number of choices than the KPS scale [6]. Moderate to high concordance rates were observed for KPS (63–75%) and ECOG PS (90–92%) in this study which included patients with better-functioning scores overall [6]. Agreement among observers is often higher when the overall patient population has a good performance status compared to when the group is more heterogeneous with respect to functional performance [13]. Due to their subjective nature, both ECOG PS and KPS scales have been criticized for poor sensitivity at the lower end of each scale. This highlights the potential for inaccurate determination of PS level for patients that fall into the lower performance status range where a wide spread is observed [15]. Another study evaluated interobserver reliability for PS measurement using four different scales (ECOG PS, KPS, and two palliative care specific scales), among three different healthcare professionals in the palliative care setting. A total of 135 patients were accrued following their referral for palliative care services. The majority (93%) had malignant disease of which 78% had metastatic or locally advanced disease. This study found low absolute interobserver agreement rates between raters using both ECOG PS and KPS scales (ECOG PS = 53–61%; KPS = 38–50%) [16].

Performance status is usually assessed by clinicians rather than patients themselves. Studies have shown only a moderate level of agreement between physician and patient recorded PS. A study looking at 206 patients with advanced lung cancer compared PS recorded by the patient, their oncologist, and nurses. There was a significant difference in PS levels recorded by all of the three groups (*p* < 0.001). Oncologists and nurses were more likely to record healthier PS levels than patients themselves [17]. Interobserver agreement of only 50% between patient and oncologist assessed PS scores was reported in another study of 98 outpatients with primary lung cancer [8].

In relation to clinical trial eligibility significant weight is often placed on PS assessment despite the high levels of interrater variability. The inability to retrospectively verify or confirm the accuracy of PS assessment represents another limitation of the standard measurement tools of PS. This limitation has been highlighted by two PS 2 focused trials in patients with advanced non-small-cell lung cancer (NSCLC) where a difference in median overall survival (OS) was observed depending on the patient's geographic location [18]. In the Selected Targeted Efficacy in Lung Cancer to Lower Adverse Reactions 3 (STELLAR 3) trial comparing carboplatin with conventional paclitaxel versus paclitaxel poliglumex in patients with PS 2, patients enrolled from the United States, Western Europe, or Canada demonstrated a median OS of 6.5 months, while those enrolled from Eastern Europe had a median OS of approximately 9 months [19]. Eastern European advanced lung cancer patients enrolled on another trial exploring paclitaxel poliglumex versus monotherapy with gemcitabine or vinorelbine also experienced a conspicuously higher median OS than that observed in patients enrolled from the United States, Canada, and Western Europe [20]. The Eastern European patients demonstrated an unprecedented survival for PS 2 patients, more in line with the outcomes expected for fit patients with advanced NSCLC. Though unexplained by the obvious demographic difference, the systematic difference in median OS among patients sharing the same eligibility features highlights that interpretation of PS represents an obvious opportunity for subjectivity and the introduction of selection bias that cannot be confirmed in retrospect as the cause for disparities in trial outcomes.

## 5. Alternative Instruments Measuring Functional Performance and Fitness for Treatment in Cancer Patients

In the area of geriatric oncology dedicated research was conducted with the aim to develop additional tools to assist the oncologist in making chemotherapy recommendations for the older person with cancer.

Older patients have the potential to derive benefit from chemotherapy similar to that derived by younger adults [41]. However, increasing age is inversely associated with recommendation and receipt of chemotherapy due to concern regarding treatment tolerability [42, 43]. For many older patients with competing causes of death the goal of treatment needs to extend beyond survival to include gains in quality of life (QoL), symptom control, and preservation of function. Therefore reducing the potential for toxicity is crucial. The existing oncology PS assessment tools previously described were validated in younger patients and do not adequately address the heterogeneity in the aging process [44]. A comprehensive geriatric assessment (CGA) is an evaluation of an older patient examining multiple domains including functional status, comorbid medical issues, nutritional status, cognition, psychological state, social support, and review of medications [45]. The variables of the CGA, particularly the domains that assess instrumental activities of daily living and comorbidities, can predict morbidity and mortality in older patients with cancer [46]. CGA has been shown to provide substantially more information regarding functional impairment of an older oncology patient than PS measurement alone [47]. Serial CGA during cancer treatment may also provide information regarding the short- and long-term effects of cancer therapy on physical function or other geriatric domains. CGA has been found to impact cancer treatment decision-making in up to 50% of cases [48]. Geriatric screening tools have been developed that increase the feasibility and speed of integration of CGA in the oncology setting [49].

Acknowledging the limitations of PS measurement alone in determining the functional status of an older cancer patient being considered for chemotherapy and the substantial additional information that CGA provides, two different risk stratification schemas were devised, the CARG and CRASH risk scores. These schemas incorporate geriatric and oncologic correlates of vulnerability to chemotherapy toxicity and help oncologists determine older patient's suitability for chemotherapy by calculating their risk of chemotherapy toxicity.

The CARG score was developed and published in 2011 by the Cancer and Aging Research Group. This study prospectively monitored 500 patients, aged ≥65 years, with stage I to IV cancer of various types through their chemotherapy course for treatment toxicity. A predictive model for grade 3 to 5 toxicity identified 11 risk factors including age, tumor type, treatment, laboratory values, and geriatric assessment variables. A scoring system based on these risk factors was devised that has the ability to categorize patients into low, intermediate, and high risk of chemotherapy toxicity. The predictive capability for chemotherapy toxicity of physician-rated KPS was inferior to the CARG score (ROC of the model with KPS was 0.53 versus 0.72 for the CARG score). No significant difference in incidence of toxicity was observed across the KPS-based risk groups (*p* = 0.19) [44].

The chemotherapy assessment scale for high age patients (CRASH) score is another predictive tool for chemotherapy toxicity in patients ≥70 years. Similarly this score was developed from a prospective study of >500 older cancer patients starting chemotherapy. Severe toxicity (defined as grade 4 hematologic or grade 3/4 nonhematologic toxicity according to version 3.0 of the Common Terminology Criteria for Adverse Events) occurred in 64% of the study population. The CRASH score consists of two subscores, one that predicts for hematologic toxicity and another for nonhematologic toxicity, each comprised of a number of clinical and laboratory variables. Similar to the CARG study a scoring system based on these variables was created that stratifies patients into low, medium low, medium high, and high risk groups for chemotherapy toxicity [50].

Other assessments of functional performance status in medicine exist. These measurement tools include the Timed Get Up and Go Test [21], the Frailty Index [22], and the Short Physical Performance Battery [23] (Table 1). They have primarily been investigated in older adult populations in whom they predict for adverse outcomes. These tools have occasionally been examined in elderly-specific cancer populations [51, 52]. For example, the ability of SPPB to predict mortality among cancer survivors was recently reported [24]. However, the use of these tools and the significance of their findings in a general oncology population are unknown.

The 6-minute walk test (6MWT) has been shown in a wide range of clinical settings to be a robust predictor of mortality [53, 54]. However, in cancer it does not appear to be a marker of prognosis. In the setting of patients with recurrent primary malignant gliomas the 6MWT was shown to provide an objective measure of physical functioning and significantly correlated with KPS and QoL [55]. Its prognostic value was later explored in the same clinical setting and functional capacity was not an independent predictor of prognosis. Exercise behavior (metabolic equivalent (MET), hours/week) assessed by a self-administered questionnaire was found to be a strong independent predictor of survival that provided incremental prognostic value to KPS [56]. In the preallogeneic hematopoietic cell transplantation setting for hematological malignancy the 6MWT was significantly associated with overall survival but again it did not provide prognostic information beyond that of traditional prognostic markers including KPS [57].

## 6. Instruments That Objectively Measure Functional Performance

### 6.1. Accelerometers

The first accelerometer was developed in the 1980s [58]. During the early years of research and development accelerometer-based devices were associated with limitations including high cost, reliability, calibration, and validity concerns [59]. Technical improvements in the monitor hardware have enabled the manufacture of accelerometers with larger memory and battery capacities, wider acceleration range, better linearity, smaller size, waterproof exterior, and lower cost [60]. These devices capture large volumes of acceleration signal data that provide opportunities to improve functional activity characterization but also present logistical and analytic challenges. These challenges are being overcome by a shift in analytic approaches from count-based approaches and linear regressions for predicting energy expenditure (EE) to functional activity characterization and EE estimation based on raw-data based analytic models [60]. Improvement in accelerometer technology has resulted in increased feasibility and compliance with accelerometer use as demonstrated by research studies such as the national health and nutrition examination survey (NHANES) [61].

### 6.2. Electronic Activity Monitoring Systems

New consumer-based activity monitors designed for use by individuals with an interest in health, fitness, and weight control are now commercially available. This technology development was driven by a number of factors including (i) increased availability of low-cost accelerometer technology in the marketplace, (ii) refinement of other technologies (e.g., Bluetooth), and (iii) the increased sophistication of personalized social media applications [62]. Electronic activity monitoring (EAM) systems are defined as wearable devices that objectively measure functional activity and can provide feedback, beyond the display of basic activity count information, via the monitor display or through a partnering application to elicit self-monitoring of activity behavior [25]. They measure the number of steps taken and/or the amount of time spent performing activities at different intensities. Activity monitors can convert basic functional activity measurements using algorithms into distance covered or number of calories burned. EAM systems improve upon the traditional accelerometer by having the ability to provide visual feedback on activity progression, verbal encouragement, and social comparison via a mobile application or a website. EAM systems are currently commonly used as “fitness trackers” to measure fitness and promote weight loss. The potential of wearable EAM devices to promote health behavior change and engage less motivated individuals has been recognized by some healthcare organizations, employers, insurers, and clinicians. Commercially available EAM systems are growing in popularity. An estimated 3.3 million fitness trackers were sold in the USA in 2014 generating over $200 million in sales [25]. Annual sales are projected to increase to more than $50 billion by 2018 [63]. This has ignited interest among researchers and practitioners to explore the feasibility, efficacy, and potential of EAM systems in the healthcare arena.

In order for this new technology to expand into the healthcare industry, EAM systems will need to innovate along the following three axes: (i) functionality, (ii) reliability, and (iii) convenience. The healthcare market and the field of clinical research demand accurate performance and valid data to inform clinical decisions. Studies exploring the accuracy of available EAM devices exist and are reviewed in Table 2  [28–32].

These studies overall confirm the reliability of some commercially available EAM systems. However, the majority of studies evaluating the reliability and validity of these systems were conducted on healthy volunteers. The algorithms used to analyze the sensor data and identify step count in EAM systems are often developed from healthy individuals with faster gait speed than frail cancer patients. This highlights the importance of exploring the accuracy of these devices in cancer patient populations.

Participant acceptability and the feasibility of EAM device use to measure physical activity have also been explored in healthy volunteers. For example, a 12-week pilot study was conducted to evaluate the feasibility of using mobile wristband activity trackers in 34, community dwelling, older adults with a median age of 73.5 ± 9.4 years. The study reports that patients found the devices easy to use with minimal study withdrawals noted [64]. Another study evaluated the feasibility of integrating a FitBit tracker and website into a physical activity intervention in 51 inactive postmenopausal women in the setting of a randomized controlled trial. Compliance with wearing the FitBit device was 95%. The entire population (100%) reported that they liked the tracker device [65]. Studies examining patient acceptability with EAM device use are required in patient populations to confirm feasibility of incorporating these technologies into the healthcare setting.

The potential of EAM devices as measurement tools of patient physical activity has been explored in various disease settings to date including gynecology patients with polycystic ovarian syndrome [66], obese patients with prebariatric surgery [67], patients with chronic diseases such as chronic obstructive pulmonary disease [68] and chronic kidney disease [69], and psychiatry patients with depression [70]. Much of the literature available on EAM systems focuses on their efficacy and feasibility as a modality within a physical activity intervention for adults. Although these studies are heterogeneous in their design and outcome measurements, they have demonstrated the ability of EAM systems to increase physical activity [71–73] and reduce weight when used as a physical activity intervention [73–76].

The limited data available on use of EAM systems in an oncology population supports the feasibility and patient acceptability of incorporating EAM devices into clinical studies [26, 27, 77]. Patient acceptability was almost 100% in a study that examined functional activity for one week using an EAM device in sixty patients with lung or upper gastrointestinal cancer. The mean compliance in this study was 98% (94–100%) [27]. Although accuracy of EAM devices has been confirmed in other patient populations there is a paucity of studies examining the reliability and validity of EAM captured data in a cancer population [35]. One study did raise concern regarding the ability of accelerometers to measure step count accurately in frail patients with advanced cancer and reduced PS. This study evaluating the validity of the accelerometer-based monitoring system reported that the device was accurate in providing estimates of body positions and transfers but not step count particularly in non-self-caring patients (KPS 40–60) compared with self-caring patients (KPS 70–100) (33% versus 24%, *p* = 0.06) [36].

Table 3 conveys cancer-specific studies incorporating EAM measured physical activity data. Descriptive studies [33, 34], correlative studies [26, 37, 38], and randomized trials [39, 40] have explored the role of EAM captured physical activity data in cancer patients and survivors. In the interventional randomized trials the EAM devices were primarily used to motivate physical activity adherence and determine the effectiveness of functional activity interventions on EAM measured physical activity levels. Interestingly one correlative study has shown in advanced lung cancer patients that a significant association exists between EAM measured physical activity level and ECOG PS. EAM recorded step count differed significantly between ECOG PS categories and step count decreased with decline in PS level [26].

## 7. EAM Systems: Potential Role in Healthcare and Oncology Care

These emerging technologies have the potential to be applied in various oncology settings. (1) Primary prevention: studies have shown that an active lifestyle reduces cancer risk [78]. These devices could be used to promote health behavior change and weight reduction, by encouraging exercise, healthy diet, and sleeping patterns. These devices may also help to develop patient self-care and management skills. (2) Cancer treatment decision-making: EAM devices may provide the means to reliably record patient physical activity levels. This method has the advantage of prolonged measurement that can be repeated at various stages throughout the patient's course of management. EAM devices may be able to provide an objective assessment of functional performance that can be retrospectively confirmed. Independently or in combination with standard measurements of PS it may provide a more accurate assessment of functional status, which could lead to better decision-making at the time of cancer diagnosis and throughout the course of cancer management. In the surgical oncology setting, EAM may be able to strengthen the accuracy of preoperative evaluation. In the immediate postoperative time, it may be able to help identify those at risk for hospital readmission or short-term mortality. In the long postoperative follow-up, EAM devices may be able to assist the timing of full functional recovery of cancer patients. In the medical oncology setting, EAM data along with other standard measurements may be able to help oncologists decide between single and multiple agent chemotherapy regimens for each patient. The continuous data may provide insight into real fluctuation in the activity level of cancer patients during the course of treatment and its relationship with chemotherapy toxicity and/or healthcare utilization. In the cancer survivorship, the EAM can be useful in promoting healthy behavior and active lifestyle. In all of these contexts, EAM has the potential to provide an objective assessment of patients' functional activity that may be more accurate than clinician-rated performance status of patients and even patient-reported data. Finally, EAM captured data may be useful in assuring that performance status of patients enrolled in clinical trials is recorded accurately. In order for the potential role of EAM devices in healthcare and oncology care settings to be realized substantially more clinical research incorporating this technology is required.

## 8. EAM Systems: Potential Challenges for Healthcare and Oncology Care

EAM devices have the potential to offer positive health-related opportunities to patients; however their introduction into the healthcare arena is not without difficulty. The potential challenges that these new innovations pose to the healthcare industry include the following. (1) Increased demand on resources: this new technology provides an abundance of data that requires processing and may strain already limited computer information systems that currently operate in healthcare settings. (2) Method of data visualization: how to present the information captured by EAM systems in a meaningful way that can be easily communicated to and interpreted by all interested parties including patients, physicians, allied health professionals, and researchers represents a challenge. (3) Cost and accessibility: the routine use of EAM devices in patient management represents an additional healthcare expenditure that may result in inequitable patient access. (4) Formal food and drug administration approval or licensing for use in a healthcare setting: if EAM systems are incorporated into routine patient care and used to guide clinical decisions they will need regulatory approval to obtain a license for use in a healthcare setting outside of the realm of clinical research. (5) Privacy and data security: valid concerns about patient privacy and data security exist in relation to use of EAM systems in the healthcare setting. It is possible that patient data and communications could be tracked. If the intention of use of these devices is to improve healthcare outcomes patients may be willing to use them despite the privacy concerns; however full disclosure to consumers regarding what is being monitored and for what purpose is important. Ultimately regulation of these devices will be required to protect patient privacy. (6) Determination of the clinical significance of EAM captured data: the potential of this innovative technology is promising; however the actual clinical significance of the information captured by EAM devices remains to be determined. Healthcare professionals are cautious about using these devices without hard evidence that the benefits justify the time and expense it takes to use them. We rely upon clinical research to investigate their clinical significance.

## 9. Recommendations for EAM Device-Based Clinical Research in an Oncology Setting

As previously highlighted limited robust clinical data exists regarding the role of EAM systems in an oncology setting. The literature available does support the feasibility of EAM device use to measure physical activity in a cancer population. However, further clinical studies assessing the feasibility and patient acceptability of this technology in an oncology population would be valuable. Further research exploring the accuracy of EAM captured functional activity data in cancer patients would also be helpful. This information would help to clarify the ability of EAM systems to provide an objective measurement of patient physical activity levels.

The next research endeavor focusing on EAM use in an oncology setting is to determine the clinical value of these devices by exploring their predictive and prognostic capabilities with regard to cancer outcomes. The objective should be to determine if EAM measured physical activity data provides clinically meaningful information that outweighs the cost of incorporating this technology into routine cancer care. In order to determine if EAM measured physical activity data can be used as a measurement of functional PS in cancer patients the next step is to determine if an association exists between EAM captured physical activity data and functional PS assessed using standard measurement tools including KPS and ECOG PS scales. If a positive correlation exists then the correlation between EAM captured physical activity data and other cancer-related outcomes such as treatment tolerability and survival should be explored. Finally, a study that examines whether an intervention introduced on the basis of information provided by an EAM system positively or negatively affects patient outcome should be considered. Identifying whether EAM captured physical activity data provides additional prognostic and predictive information beyond that provided by standard measurements of PS will determine whether EAM measured physical activity data should be incorporated into routine cancer care and clinical trial designs. Studies incorporating cost benefit analyses will also help to determine whether using EAM systems in an oncology setting is worthwhile.

## 10. Conclusion

Significant emphasis is appropriately placed on the measurement of functional PS of a cancer patient. However, the standard tools used by the oncology community to measure PS are associated with limitations that stem from the subjective nature of these assessments. Development of an objective measurement instrument would improve the ability to accurately and reliably determine patient PS. Ultimately this would lead to better patient outcomes.

An EAM system is a new technology that has become increasingly popular among consumers as a fitness tracker that aims to promote behavioral and lifestyle changes. These devices may be able to provide accurate and objective measurements of functional activity. EAM devices have significant potential for use in the healthcare setting most notably as an objective assessment of functional PS in cancer patients. Well-designed clinical research incorporating this technology is imperative. Clinical trials are required to determine if EAM measured physical activity represents an accurate and objective assessment of functional performance that provides predictive and prognostic information incremental to that already provided by the standard measurement instruments of PS. The clinical significance of the information captured by EAM systems relevant to the cancer patient is currently unknown and must be determined through completion of high quality clinical research. Accurate measurement of patient fitness for treatment would help oncologists select the most appropriate treatment options for patients that would in turn reduce toxicity and improve patient outcomes.

## Figures and Tables

**Table 1:** Alternative measurement instruments of functional performance.

Assessment tool	Description	Nature of assessment	Feasibility	Limitations	Correlation with outcomes
KPS [3]	Linear scale from 0 (dead) to 100 (normally active, without evidence of disease) summarizing ability to perform daily activities, and level of assistance required	Subjective	Time to completion: minutes Easy to perform No equipment required	Poor reliability, variable levels of interobserver agreement Subjective, poor concordance between patient recorded and physician recorded KPS Poor predictor of prognosis when PS is good Poor sensitivity at the lower end of the scale Validated in a younger patient cohort and less applicable to an older heterogeneous patient group Unable to verify accuracy of assessment	Survival Prognosis Response to chemotherapy Chemotherapy toxicity Quality of life

ECOG PS [5]	6-point scale ranging from 0 (fully active) to 5 (dead), assessing level of function, ambulation, and capability of self-care	Subjective	Time to completion: minutes Easy to perform No equipment required	Poor reliability, variable levels of interobserver agreement Subjective, poor concordance between patient recorded and physician recorded KPS Poor sensitivity at the lower end of the scale Poor predictor of prognosis when PS is good Unable to verify accuracy of assessment	Survival Prognosis Response to chemotherapy Chemotherapy toxicity Quality of life

TUG [21]	Assessment of mobility and functional balance; functional mobility is quantified by the time it takes a patient to get up from a seated position, walk 3 meters, turn around, and return to a seated position	Objective	Time to completion: seconds Easy to perform Minimal equipment Required	Focused on one domain, mobility	Risk of falling Gait speed Residential status Ability to go outside alone safely Berg Balance Scale Barthel Index of Activity of Daily Living

Frailty Index (FI) [22]	FI is the ratio of the deficits present in a person to the total number of potential deficits evaluated	Subjective and objective components		Expensive	Mortality Predicts risk of adverse health outcomes

SPPB [23, 24]	SPPB measures physical and functional health and consists of three subtests: 4-meter walk, repeated chair stands, and standing balance	Objective	Time to completion: 5–10 minutes More complex assessment, 3 tests Minimal equipment required	Cost Inconvenient	Mortality (data available for older cancer patients and survivors)

EAM [25–27]	Wearable devices that objectively measure physical activity and can provide feedback	Objective	Time to completion: continuous assessment, duration dictated by patient Requires expensive equipment Feasibility and patient acceptability have been demonstrated	Cost Reduced accessibility Increased demand on healthcare computer information systems	Physical intervention, weight reduction Correlation with ECOG PS

KPS: Karnofsky Performance Status, ECOG PS: Eastern Cooperative Oncology Group, TUG: Timed Get Up and Go, SPPB: Short Physical Performance Battery, EAM: electronic activity monitoring, and PS: performance status.

**Table 2:** Accuracy of objective activity monitoring devices.

Reference	Population	#	Age (yr)	Device under study	Activity monitored	Method of confirmation	Outcome
[28]	Healthy volunteers	14	Mean (SD) = 28.1 (6.2)	Pedometer, Digi-Walker SW-200 (Yamax) Accelerometer, Zip and One (Fitbit) Wearable devices, Flex (Fitbit), UP24 (Jawbone), FuelBand (Nike) Mobile apps: 3iOS, Fitbit, Health Mate (Withings), Moves (ProtoGeo Oy) Android app: Moves (ProtoGeo Oy)	Walking 500 and 1500 steps @ 3MPH	Observer counted steps using tally counter	High accuracy confirmed for all devices

[29]	Healthy volunteers	15	Range 21–33	Wearable device, Fitbits (One, Zip, and Flex)	6 activities for a 5-minute period @ 1.5, 3, and 4 MPH	Video analysis	90% accuracy for all devices at all speeds

[30]	Healthy volunteers	89	Mean (SD) = 39 (±13.1)	Lumoback, Fitbit Flex, Jawbone Up, Nike+ FuelBand SE, Misfit Shine, Withings Pulse, Fitbit Zip, Omron HJ-203, Yamax Digi-Walker SW-200, and Moves mobile application versus Optogait system for laboratory and ActivPAL for free living conditions	(A) Laboratory setting: walked twice on a treadmill (4.8 km/h) for 30 min (B) Free living conditions, physical activity over a 24 hr period	(A) Laboratory setting, Optogait system (B) Free living conditions, ActivPAL	Good reliability for step count, Lumoback, Jawbone Up, Misfit Shine, Withings Pulse, Fitbit Zip and Flex, and Digi-Walker Free living conditions, Fitbit Zip highest validity, Nike+ FuelBand, lowest validity

[31]	Healthy volunteers	21		Fitbit One, Fitbit Zip, Jawbone Up, Misfit Shine, Nike FuelBand, Striiv Smart Pedometer, and Withings Pulse	48 hr of PA in free living conditions	Research grade accelerometers: BodyMedia SenseWear and ActiGraph GT3X+	Strong validity between research grade accelerometers and all activity monitors for step count and sleep duration Moderate validity for MVPA and TDEE Strongest performers, Fitbit One, Fitbit Zip, and Withings Pulse

[32]	Patients with stroke and traumatic brain injury	50	Mean (SD) = 52.9 (±15.1)	Fitbit Ultra and Nike+ FuelBand	Two-minute walk test (2MWT)	Video analysis and research grade activity monitors: StepWatch Activity Monitor (SAM) and Yamax Digi-Walker SW-701 pedometer (YDWP)	In order of decreasing accuracy, SAM, Fitbit Ultra, YDWP, and Nike+ FuelBand

yr: year, MPH: miles per hour, hr: hour, PA: physical activity, MVPA: moderate to vigorous physical activity, and TDEE: total daily energy expenditure.

**Table 3:** Clinical studies incorporating EAM systems in cancer populations.

Reference	Sample, *N* = number of participants	Device	Study design	Study objective	Results relevant to EAM measured PA data
	Descriptive and feasibility studies				
[33]	Breast cancer survivor *N* = 398, noncancer controls *N* = 1120	ActiGraph Accelerometer: survivors, Model GT1M, controls, Model AM-7164	Case control study	Compare level of PA and sedentary behavior between survivors and controls	Breast cancer survivors are more sedentary than noncancer controls
[34]	Cancer survivor, *N* = 7,285,825, noncancer controls, *N* = 162,502,859	ActiGraph AM-7164	Case control study	Compare PA levels among cancer survivors and controls	Neither group met the CDC guidelines for physical activity, survivors, 95.5% controls, 87.3%
[27]	Lung and upper gastrointestinal cancer patients (ECOG PS 0–2) *N* = 60	ActivPAL	Prospective, observational study	Assessment of patient acceptability using compliance based on analysis of movement data	EAM device fulfilled the definition of acceptability; 98% wore the EAM ≥80% of the time

	Reliability/accuracy of EAM devices				
[35]	Hematologic malignancy Patients, *N* = 23, healthy controls, *N* = 30	Step Accelerometer 3 (SAM3)	Prospective, case control study	Compare reliability of the assessment of PA and compare PA levels between patients and controls	Device, good reliability Cancer patients, significantly less active than healthy subjects (5355 versus 6364 steps, *p* ≤ 0.05)
[36]	Advanced cancer patients (KPS 40–100), *N* = 56, healthy volunteers, *N* = 9	ActivPAL	Prospective, observational study	EAM measured PA data was validated against video-recorded observations and EE measured by DLW protocol	Step count error higher in patients with KPS 40–60% versus KPS 70–100% (33 versus 24%, *p* = 0.006)

	Correlative studies				
[37]	Breast cancer patients, *N* = 32, noncancer control, *N* = 30	ActiGraph Model GT3X	Prospective, case control study	Examine the effect of PA level on working memory	Greater PA level was positively and significantly associated with better working memory performance across both arms (*p* = 0.014)
[38]	Colon cancer survivors *N* = 180	ActiGraph Model GT3X	Cross-sectional, observational study	Determine the association of EAM measured MVPA with QoL and physical functioning	MVPA was significantly associated with better QoL (*p* = 0.038) and physical function (*p* = 0.009)
[26]	Incurable thoracic cancer patients (ECOG PS 0–2) *N* = 84	ActivPAL	Retrospective study	Determine the correlation between EAM measured PA and ECOG PS	Decline in ECOG PS was significantly associated with decline in EAM measured step count (*p* < 0.05)

	Interventional studies				
[39]	Breast cancer survivors (stages I–IIIA) *N* = 377	Pedometer Digi-Walker SW-200 (home-based; 12 weeks; 30 minutes, 5 days a week, ≥moderate intensity)	RCT Arm 1: standard public health recommendation Arm 2: breast cancer specific print materials Arm 3: pedometer Arm 4: combination of print materials and pedometer	Determine the effects of breast cancer-specific PA print materials, pedometer, or their combination, on self-reported PA and QoL in breast cancer survivors	Significant intervention effect on self-reported physical activity and walking but no intervention effect on daily steps. Effect seen in all interventional arms. Combination arm, significantly improved QoL (*p* = 0.003)
[40]	Breast and prostate cancer patients receiving radiation therapy *N* = 38	Pedometer, details not reported Home-based; 4 weeks; moderate intensity walking (10,000 steps/day); resistance bands daily	RCT Arm 1: exercise intervention Arm 2: no exercise intervention	Examine the feasibility and initial efficacy of a home-based aerobic and progressive resistance exercise intervention for aerobic capacity, strength, muscle mass, CRF, and QoL	Significantly greater increase in daily steps, higher QoL, and lower CRF in intervention group after intervention and at 3-month follow-up.

EE: energy expenditure, DLW: doubly labeled water, EAM: electronic activity monitor, MVPA: moderate to vigorous intensity physical activity, QoL: quality of life, PA: physical activity, RCT: randomized controlled trial, and CRF: cancer-related fatigue.
